# Commercialisation of CMOS Integrated Circuit Technology in Multi-Electrode Arrays for Neuroscience and Cell-Based Biosensors

**DOI:** 10.3390/s110504943

**Published:** 2011-05-04

**Authors:** Anthony H. D. Graham, Jon Robbins, Chris R. Bowen, John Taylor

**Affiliations:** 1 Department of Electronic & Electrical Engineering, University of Bath, Bath, BA2 7AY, UK; E-Mail: j.t.taylor@bath.ac.uk; 2 Receptors & Signalling, Wolfson CARD, King’s College London, London SE1 1UL, UK; E-Mail: jon.robbins@kcl.ac.uk; 3 Department of Mechanical Engineering, University of Bath, Bath, BA2 7AY, UK; E-Mail: c.r.bowen@bath.ac.uk

**Keywords:** IC, CMOS, biosensor, biocompatibility

## Abstract

The adaptation of standard integrated circuit (IC) technology as a transducer in cell-based biosensors in drug discovery pharmacology, neural interface systems and electrophysiology requires electrodes that are electrochemically stable, biocompatible and affordable. Unfortunately, the ubiquitous *Complementary Metal Oxide Semiconductor* (CMOS) IC technology does not meet the first of these requirements. For devices intended only for research, modification of CMOS by post-processing using cleanroom facilities has been achieved. However, to enable adoption of CMOS as a basis for commercial biosensors, the economies of scale of CMOS fabrication must be maintained by using only low-cost post-processing techniques. This review highlights the methodologies employed in cell-based biosensor design where CMOS-based integrated circuits (ICs) form an integral part of the transducer system. Particular emphasis will be placed on the application of multi-electrode arrays for *in vitro* neuroscience applications. Identifying suitable IC packaging methods presents further significant challenges when considering specific applications. The various challenges and difficulties are reviewed and some potential solutions are presented.

## 1. Introduction

The definition of a ‘biosensor’ as stated by the IUPAC ius ‘*a device that uses specific biochemical reactions mediated by isolated enzvmes, immunosystems, tissues, orcranelles or whole cells to detect chemical compounds usually by electrical, thermal or optical signals.*’ [1]. The progress and success of biosensor development therefore requires a highly multi-disciplinary approach and a single application may require leading-edge contributions from neuroscientists, biologists, semiconductor engineers, electronic hardware designers, pharmacologists and surgeons.

**Figure 1.**
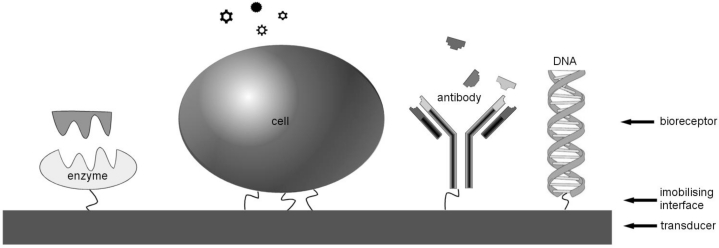
Elements of a biosensor. Various biological elements may form the bioreceptor which are immobilised on the transducer (adapted from [3]).

Furthermore, most functional sensors consist of two parts: first a biological receptor or *bioreceptor* that detects the presence of the substance under test (the *analyte*) and, secondly, a *transducer* that detects a response of the bioreceptor to the analyte and translates this into an output signal. The sensing bioreceptor is usually immobilised on the chemical/physical transducer either by some natural adhesion processes or by coating the surface [2]. The bioreceptor typically comprises either cells, DNA, enzymes or antibodies, as illustrated in Figure 1.

**Figure 2.**
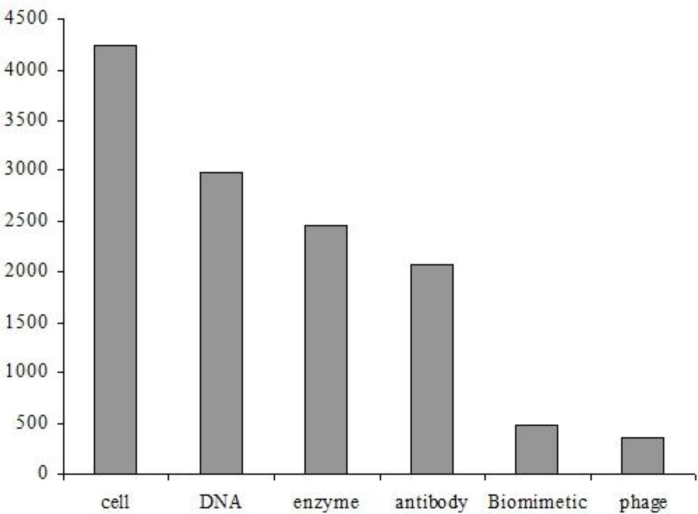
Analysis of the biosensor literature containing the terms ‘CMOS’ or ‘integrated circuit’, (data source: ISI Web of Knowledge and Google Scholar, 3rd February 2010).

Transducers can broadly be classified into three groups, namely optical, electrochemical and mass-based detection methods. Of most interest in this review are the electrochemical techniques that are more relevant to *integrated circuit* (IC)-based biosensors and the packaging technologies that form an integral part of such a device. Figure 2 provides an analysis of the relevant biosensor literature containing the terms ‘CMOS’ or ‘integrated circuit’. It is clear that *cell-based* types are the most popular in practice. This fact together with the authors’ specific interest in IC-based biosensors led to the choice of this as the main subject of this review.

One of the earliest publications to define and discuss biosensors was ‘*Biomedical Telemetry*’ in 1965 [4]. At this time, ICs were in their infancy and a patent for a *Complementary Metal Oxide Semiconductor* (CMOS) had only recently been granted [5]. Subsequently, this technology has become the undoubted foundation of modern electronics and now dominates the worldwide IC market. Economies of scale have made CMOS very cheap and easily available and perhaps, therefore, naturally attractive to the designers of many types of biosensors. However, due to the specific materials available in a CMOS process (specifically aluminium and its oxide), the use of this technology to form a transducer raises the problem of the analyte/electrode interface and potential issues of neurotoxicity [6]. This is generally considered to be the main roadblock to CMOS biosensor commercialisation at present. In fact, it can be argued that CMOS technology has not yet penetrated the commercial market for these devices. Consider, for example, the use of *multiple electrode arrays* (MEAs, The term ‘*micro*-electrode array’ refers to the same technology and is similarly abbreviated to MEA) for *in vitro* neuronal recordings. The MEA represent the transducer element described above. A MEA becomes a biosensor if a bioreceptor is attached to the MEA and acts as a sensing element. MEAs have been developed as a transducer for direct interfacing with brain slices or dissociated neurons. Commercial MEAs are passive components that are custom-fabricated, expensive, have short lifetimes and have no ability to process the recorded signals. Because of these shortcomings, analysis of the literature confirms there is significant interest in making the electrode more intelligent by marrying integrated circuit technology with cell-based biosensors, which logically leads to the use of CMOS in this application.

In summary, this review will initially cover the field in a general manner before focusing on CMOS MEAs for *in vitro* neuroscience applications. The material is structured as follows. Section 2 provides an overview of transducers as applied to biosensors in general, before refining the discussion to CMOS types with particular reference to manufacturability. Section 3 presents an overview of CMOS technology. Section 4 discusses the important role of the metal surface and metal-solution interface which are of general interest to researchers interested in the use of CMOS transducer elements in biosensor applications. Section 5 reviews a most demanding application, namely neuronal interfaces and details some practical aspects required for successful neuronal recordings. Section 6 reviews the all-important area of packaging technology and Section 7 discusses obstacles to commercialisation. The review concludes with an overview of likely further work in this field.

## 2. Transducers

As already noted, *transducers* can broadly be classified into three groups, based on optical, electrochemical and mass-based detection methods. Of most interest in this review are the electrochemical techniques that are more relevant to IC-based biosensors. *Amperometric* transduction [7] is a current-measuring sensor using two electrodes, operated at constant potential and is highly sensitive to the concentrate of an analyte. *Conductometric* transducers operate in a similar manner by detecting changes in the electrical conductance of an analyte [8]. Similarly, *potentiometric* detection comprises a measure of electric potential at zero current (known as the ‘open circuit potential’ or OCP) and varies logarithmically with analyte concentration, thereby allowing the detection of very small changes in concentration [9]. However, it may be noted that the distinction between amperometric, conductometric and potentiometric methods is largely historical [10] and all approaches can be integrated using CMOS circuits adjacent to the transducer. An extension of these methods is the light-addressable potentiometric sensor (LAPS) which detects changes in the junction potential of a doped silicon layer when subjected to a photocurrent produced from an external light source [11,12]. An impedimetric (impedance) transducer is another form of electrochemical sensor that can be used in the label-free measurement of viable cells [13]. Piezoelectric materials can be used as resonance mass-based transducers by detecting the additional mass of chemicals binding to the surface, for example coated with an antibody or other bioreceptor [14]. The frequency of piezoelectric crystal oscillation varies with applied mass which can then be detected electrically (Quartz Crystal Microbalance—QCM, Surface Acoustic Wave—SAW). Lastly, biosensor transducers using the magnetoelastic properties of ferromagnetic materials can also be used to detect changes in mass when they are used as resonating micro-cantilevers [15].

### 2.1. Transducer Suitability for CMOS Manufacture

Confirmation that the electrode interface is the main roadblock to CMOS biosensor commercialisation at present, has been corroborated in [16], where they state:

‘The primary design challenge using CMOS technology is the interface design between assay and integrated chip (IC) which generally calls for additional post-fabrication steps to facilitate compatibility in detecting targets (e.g., analytes).’

Constructing an overall picture of biosensor research is impeded by the large quantity of published articles and patents combined with the diversity of the technologies and applications. However, a quantitative analysis of the literature shows the most commonly reported IC-based biosensors use cells as bioreceptors with optical fibre or piezoelectric transducers. The fibre optic element is not a true transducer in its own right since its role is more accurately described as a conduit to a transducer that is remote from the bioreceptor and analyte [17]; these remote sensors can use various spectroscopic techniques such as absorption, fluorescence, phosphorescence and surface plasmon resonance. Additionally, cell-based biosensors using piezoelectric, magnetoelastic or optical-based transducers [Raman, FTIR (Fourier Transform Infrared Spectroscopy), SPR (Surface Plasmon Resonance)] are not readily implemented in standard CMOS integrated circuits and therefore lie beyond the scope of this review. Conversely, electrochemical (amperometric, potentiometric, conductometric, impedimetric) transducers are most suited to manufacture using standard CMOS processes since electrodes in contact with an analyte can be readily formed on the surface of the integrated circuit (the formation of CMOS electrodes is discussed later in Section 4.3). These electrodes may be used in conjunction with the various types of bioreceptor discussed above.

The use of complex IC technology in a biosensor application naturally needs justification. Where, for example, a passive device is called for, requiring no transistors and where a single layer can define both the tracks and the electrodes, then this may often be achieved cost-effectively using a custom manufacturing process employing simple photolithographic methods. However, when a specification calls for circuitry close to the electrodes, such as low noise pre-amplifiers for neuronal recordings, the benefits of using CMOS are obvious. Research on biosensors using CMOS presently utilise mature fabrication processes, with the ability to define features of only ∼0.1 μm or larger. However, the industry is currently working toward features as small as 22 nm for 2011 [18]. The economies of scale resulting from volume manufacturing and the ability to pack data processing capabilities into very small areas of silicon chip enable multi-electrode arrays (*i.e.*, the transducer element) with excellent spatial resolution to be manufactured. These advantages are also combined with a much lower device cost than can be achieved using a custom semiconductor manufacturing process.

## 3. An Overview of CMOS Technology

CMOS is currently the dominant technology used worldwide in IC products; see Table 1 for a list of foundries. It is therefore of no surprise that research has attempted to adapt CMOS for other applications such as in biosensors. CMOS processes have always been purposely developed to be closed to the surrounding environment to avoid contamination problems that lead to low manufacturing yield and poor reliability. Therefore opening the chip surface to form a transducer is somewhat inconsistent with the goals of most semiconductor manufacturers.

**Figure 3.**
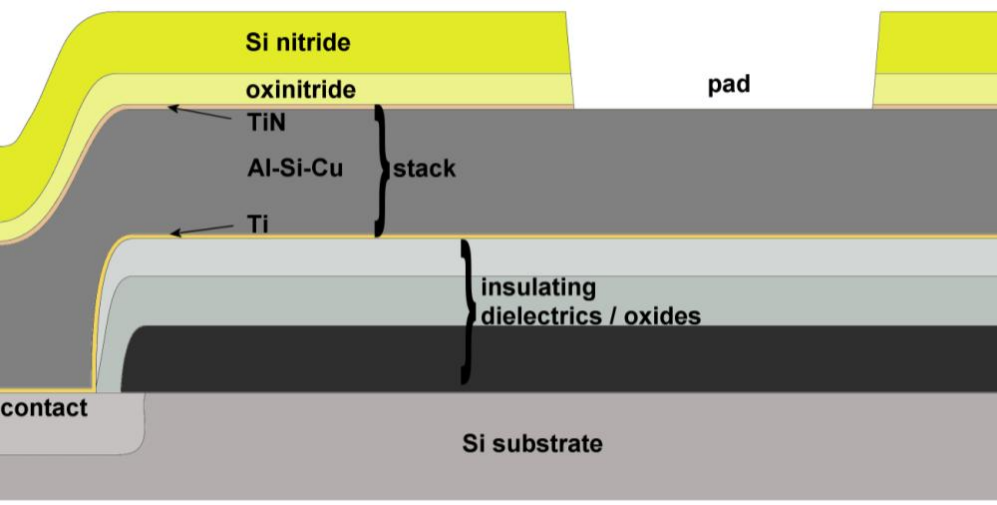
Typical CMOS metallisation (simplified, and for brevity showing only single-layer metal). The thickness of the marked ‘stack’ is typically 1 μm and here comprises a titanium barrier layer, an alloy of Al-Si-Cu and a titanium nitride anti-reflective coating.

The general structure of a CMOS IC is shown in Figure 3. A single metal layer is shown in this generic example. The transistors are formed within the silicon substrate and the transistor gates are formed above the field effect transistor (FET) channel regions. The first layer of metal is then deposited, forming contacts with the transistor source and drain regions. An *interlayer dielectric* (ILD) is deposited onto the metal to insulate the conducting layers from each other. Additional metal layers can therefore be deposited; each one insulated from the layers below using additional ILD layers. Windows in the ILD layers allow connection (*vias*) between adjacent metal layers.

Finally, a film of insulator, often comprising two separate layers, is deposited over the chip which provides *passivation* and protects the circuits from physical damage and from external contaminants. The only standard openings in the passivation are onto bondpads formed from the top layer of metal. The bondpads provide electrical connections to/from the chip. Similar openings can also be used to form metal electrodes functioning as transducers. The section shown in Figure 3 has only one metal layer, but modern CMOS processes may have six or more such layers. The processing of the various layers requires flattening of the surface between each metal deposition: these are called ‘*planarised*’ processes and avoid problems of metal and insulator coverage (‘*step coverage*’). The result of planarisation is that the chip surface is flat, with steps only at the openings of the bondpads. The height variation of passivation in the unplanarised processes may be several microns and therefore might be a consideration in the positioning of cells on surfaces of CMOS transducers [19].

*Aluminium* forms the conventional basis for high volume IC metallisation and is likely to continue to be the material of choice for the foreseeable future (for the final metal layer in a fabrication process, either as standard or as a process option). A typical metallisation stack is shown in Figure 3. The inclusion of a small proportion of copper (typically 0.5% and up to 4%) reduces the reliability problem of *electromigration* [20,21]. Due to the presence of shallow silicon junctions at contacts (where metal contacts silicon) it is also necessary to prevent ‘*contact spiking*’; namely the eutectic alloying of the aluminium and silicon. This is frequently achieved through the incorporation of a ‘*barrier layer*’ at the base of the metal stack and by alloying the aluminium with silicon—typically 1 to 2 wt%. Typical materials for barrier layers are titanium, titanium nitride and titanium-tungsten [21]. Additionally, it is frequently necessary to include an *anti-reflective coating* (ARC) on top of the stack to prevent undesirable photolithography problems (typically titanium nitride (TiN) [21], as illustrated in Figure 3).

Mature CMOS processes that are likely to be used for small quantity production, such as for MEAs, are typically >0.1 μm processes which continue to use aluminium for metallisation. For deep submicron (<0.1 μm) processes, the semiconductor industry has moved to the use of copper, but this may not be totally complete for niche applications until the 45 nm node is reached [22–24]. Even on these more advanced processes, aluminium is often used to coat the final (uppermost) metal layer to ensure high quality wire-bonding.

## 4. Practical Aspects for Successful Neuronal Recordings

As already noted, the design of cell-based biosensors is exceptionally multi-disciplinary. Clearly, an understanding of cell physiology is a pre-requisite to any consideration of cell-based biosensors. However a detailed overview of this material is beyond the scope of this review and can be studied in a variety of standard texts, e.g., [25]. Other aspects, also common to many other type of biosensor, include cell adhesion, the metal-solution interface and biocompatibility: each of these is described in some detail in the following subsections. These are also interrelated since it will be shown that a cell must be in intimate contact with an electrode for neuronal recordings, having changed its morphology from conceptually spherical and motile [motility is the ability for a cell to move (migrate) without the application of an external force] to flattened and adhered. The basic principles of CMOS technology were reviewed in the previous section. This was essential since the modification of standard CMOS is a critical current research topic if the technology is to meet the needs of a particular cell-based biosensor. This section contains a discussion of the use of CMOS MEAs for neuroscience applications. In this case the MEA can be a simple transducer element used to sense the action potentials of neurons or can be part of a cell-based biosensor if the neuron is acting as a bioreceptor (e.g., for high throughput drug screening).

### 4.1. The Aluminium Surface and Aluminium-Solution Interface

Prior to discussing the modification of CMOS electrodes and the changes necessary to satisfy the requirements of a practical transducer for cell-based biosensors, it is essential to understand the metal surface which is naturally presented by aluminium pads to environments such as physiological solutions and cells. The nature of aluminium is determined by its physical and chemical characteristics which are fundamental to its performance as an electrode material. The environments which are of most interest here for cell-based biosensors are aluminium in air, water and physiological media. Aluminium, along with other metals used in medicine such as titanium, is very reactive—its surface reacts spontaneously with air to form a ‘natural’ oxide film of amorphous Al_2_O_3_. This reactivity is determined by the Gibbs free energy of formation, being highly negative for aluminium (−791.15 kJ·mol^−1^) and titanium (−888.8 kJ·mol^−1^) [26,27]. The instantaneous reaction with oxygen in air results in an oxide growth rate that is proportional to log-time: the thickness forms very quickly to approximately 10 nm after which it is self-passivating, preventing further reaction and film growth [28].

**Figure 4.**
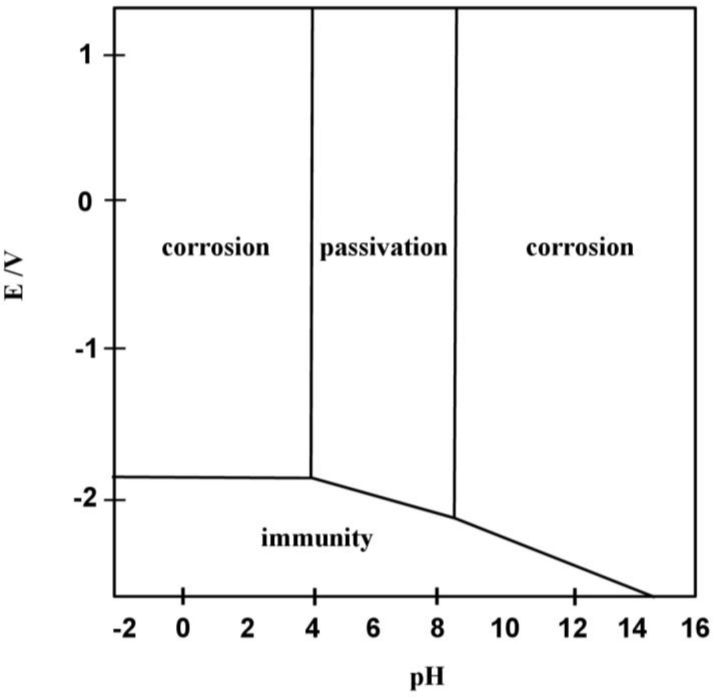
Pourbaix diagram for aluminium. This is derived from the Nernst equation and is used to show the regions of thermodynamic stability for a metal/electrolyte interface as pH *versus* electrode potential, *E*.

The stability of the metal and oxide film in a medium is defined by the electrochemistry of corrosion and is best illustrated using a *Pourbaix* diagram as shown in Figure 4. It is important to consider the pH not only of the bulk solution but also the localised conditions. The electrochemical potential, *E*, defined by the Nernst equation, will also vary due to local conditions such as alloying species, defects and contaminants. At low pH, aluminium dissolves to form Al^3+^ ions and at high pH it dissolves to form AlO_2_^−^, these conditions both being the basis of corrosion. In deionised water corrosion should not occur, but in saline physiological medium, Cl^−^ chloride ions will be adsorbed to the surface which creates localised acidic conditions (*i.e.*, dilute hydrochloric acid) under which the passivated surface deteriorates leading to corrosion of the underlying metal [29]. This process then accelerates (auto-catalytic) since the aluminium dissolution process causes a further increase in Cl^−^ concentration at the corrosion site. A saline physiological medium is therefore potentially damaging to an unprotected aluminium CMOS electrode [30].

In addition to the stability of an aluminium surface exposed to a biological medium, it is vital to understand the factors that determine the electrical characteristics of such an interface. For example and as discussed later, the electrical characteristics of electrodes for transducers sensing neuronal activity are determined primarily by the chemistry of the solid-solution interface. The basis for modern interface models originates from the theory devised by Hermann von Helmholtz in the nineteenth century, later refined by Gouy and Chapman. The basic premise of the models is the existence of an *electrical double layer* at the solid-solution interface which in turn forms the basis of electrical circuit models of the interface. These theories are presented in [27] and [31] and will not be discussed further in this review.

### 4.2. Biocompatibility of CMOS Electrodes

Any CMOS electrode in contact with biological cells, tissue and/or physiological medium needs to be compatible with its environment, must not alter the physiology of the analyte under investigation or being detected and must be non-toxic to all the biological components in the system. Unfortunately, one difficulty with assessing biocompatibility of IC materials is that no definitive list has been compiled [32] and therefore results must be taken from more loosely related applications such as orthopaedics or smaller evaluations.

Without modification of the CMOS pads, the surface presented by nearly all CMOS technologies is *aluminium.* The biocompatibility of aluminium and its oxide, *alumina*, have been thoroughly studied, much work having being done to evaluate *in vivo* performance for use with orthopaedic prosthetics [33,34]. The performance of aluminium also depends much on the adherent superficial native oxide layer (alumina) and corrosion. However, the *in vivo* use of alumina has generally been confined to orthopaedics. This is because of its poor compatibility with blood due to its thrombogenic action (*i.e.*, its tendency to cause undesirable clotting). Frequently, aluminium is coated with titanium nitride to improve *in vivo* performance [33]. For applications ranging from prosthetics to CMOS transducers, the overall *in vivo* interaction with their environment is primarily governed by the natural chemistry of the body: simplistically, this is a NaCl aqueous solution of concentration ∼0.1 M with organic acids, proteins, enzymes, macromolecules, electrolytes, dissolved oxygen and nitrogenous compounds. The resulting pH is approximately 7.2, often decreasing to ∼5.5 in the vicinity of tissue damage. Possible interaction mechanisms between any material, such as an electrode, and the body include inert/bioinert (no reaction), biodegradation (gradual breakdown by biological or biochemical processes), bioresorption (removal by cell activity or by continuous ionic diffusion) and bioactivity (a specific behaviour of a material).

The degradation of the native oxide of aluminium (alumina) on a CMOS aluminium electrode in a physiological environment is limited by its natural corrosion resistance due to the metal being in its highest oxidation state. However, a concern is that either defects in the oxide film on an electrode may enable aluminium ions to leach into the body or that the alumina itself may degrade. In physiological conditions aluminium easily forms an insoluble Al(OH)_3_ precipitate or a solution of AlCl_3_. The toxicity of these and other aluminium salts (10–100 mM) has been evaluated and shown to have only a small effect on the viability of mammalian neuronal cells [35].Additionally, neurotoxicology research into Alzheimer’s, has not shown a causal relationship between the disease and aluminium exposure [36]. Walpole *et al.* [37] and Karlsson *et al.* [38] tested nanoporous alumina substrates for aluminium ion leakage and found the dissolution rate of ions into culture measured after nine days was sufficiently low to be considered as non-toxic. In the context of IC materials, an *in vitro* assessment in [39] showed an enhanced proliferation (vitality) of Caco2 epithelial cells on aluminium *versus* the glass controls. These results therefore suggest that aluminium with a stable native oxide may form a biocompatible surface as a transducer for cell-based biosensors, but only where the electrode remains electrochemically stable—a condition usually difficult to achieve in the context of biosensors.

Whilst we are primarily interested in the CMOS electrodes in the context of this review, the biocompatibility of the surrounding material must not be overlooked. CMOS ICs predominantly use silicon nitride as the surface (passivation). Receveur *et al.* [32] concluded that silicon nitride is biocompatible, as stated by references [40–42]. In [39] it was shown that silicon nitride was an excellent substrate for Caco2 cell proliferation.

### 4.3. Practical Aspects for Successful Neuronal Recordings: The CMOS-Neuron Interface

Research into CMOS interfaces for neuronal recordings has centred mainly on two types of electrode: the electrolyte-oxide-semiconductor (EOS) FET and the metal electrode. These are explored in subsections (a) and (b) below while section (c) describes some more recent research using porous alumina based electrodes. It is maintained that the composition of the transducer interface is the primary obstacle to designing successful products; other aspects of a CMOS neuronal interface product, such as signal amplification, data processing and communication, can leverage capabilities that are already well-established in the semiconductor industry. In this respect, impressive CMOS MEAs have already been demonstrated [43–51] and so the design of CMOS circuitry is not a focus of this review.

#### (a) The Electrolyte-Oxide-Semiconductor (EOS) FET

First, an EOS FET interface has been pursued by Fromherz *et al.* at the Max Planck Institute of Biochemistry, Munich, as it offers the potential of providing a first-order (direct) response to the action potential (*i.e.*, FET current is proportional to membrane potential) [52,53]. For commercial implementation, complex (lithographic) post-processing of the IC may be required to form EOS FETs from standard CMOS processes. This is because the CMOS gate oxide layer is below the passivation and all metal layers. CMOS gate oxides are therefore not readily interfaced directly to culture medium and cells. A further concern—little emphasised in the literature—is the possibility that such an arrangement might be adversely affected by ionic contamination from contact with the culture medium [20]. Drifting of EOS FET voltage thresholds could conceivably be compensated for within an amplifier design, but ionic contamination, being highly mobile, is just as likely to cause rapid functional failures in CMOS operational amplifiers or logic gates surrounding the electrode array. This may ultimately limit the ability to use this form of EOS FET to produce a commercial product with a useful lifespan.

**Figure 5.**
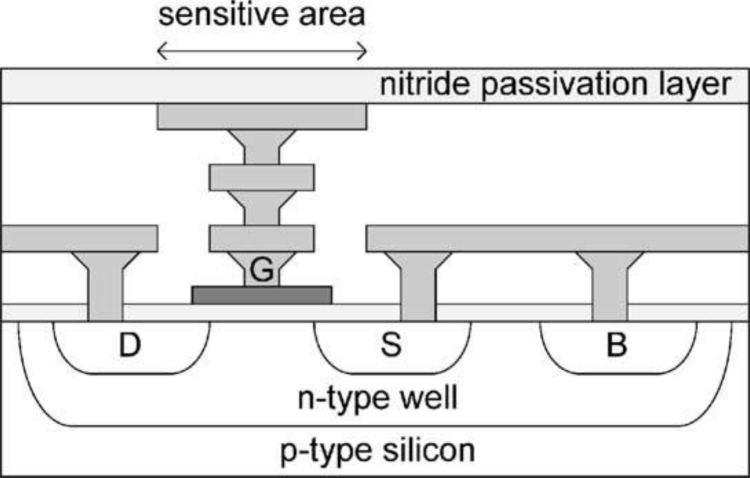
A floating gate EOS (Electrolyte-Oxide-Semiconductor) FET. The FET gate, G, is accessed from the top of the IC through the metal layers. The upper metal layer defines the sensitive (electrode) area which is covered by the silicon nitride passivation. Charge above the sensitive area induces a charge on the FET gate which in turn modulates the current in the n-type silicon channel between source, S, and drain, D. (From [56]).

An adaptation of the EOS FET has been presented in [55] and [56] which improves the passivation of the transistor by connecting the standard polysilicon gate of the sensing FET to the surface of the IC (Figure 5). The top layer of metal defines the sensor area but this is covered by standard IC passivation to avoid the need for post-processing. This process is reported to work well when the electrode is configured as an ion sensitive field effect transistor (ISFET) [55]. For cell-based sensors, sensitivity improvements have been necessary by switching the standard, thick (typically 1–2 μm) CMOS passivation to a hafnium high-κ dielectric passivation [57,58]. The hafnium process may re-introduce the need for photolithography to open windows for bondpads and therefore the cost of such post-processing needs consideration. Preliminary tests showed the thin (50 nm) hafnium film successfully passivates the aluminium pads from corrosion for short cell-based assays of five days. Further work is required to assess the integrity of the thin film during longer periods of use (for example, *in-vitro* cell cultures may be up to, say, 56 days [56] and electrodes may be expected to be re-usable). Conceivably, a thicker uniform hafnium film could be deposited over the whole device after wire bonding and assembly, but unfortunately hafnium deposition is a 250 °C process under vacuum that, whilst compatible with devices at the wafer level, is incompatible with packaged devices (moulding compounds and elastomer may out-gas or fracture under high vacuum and may decompose at 250 °C).

As a result, it seems that implementing an EOS FET in CMOS either leads to an ionic contamination hazard or requires post-processing photolithographic steps in a cleanroom to define bondpad windows in a hafnium passivation. Indeed the need for post-processing for these FETs has been confirmed in a recent review [59]: ‘With an appropriate post-process these [floating-gate FET] devices can be operated in a liquid environment.’

#### (b) The Use of Metal Electrodes

**Figure 6.**
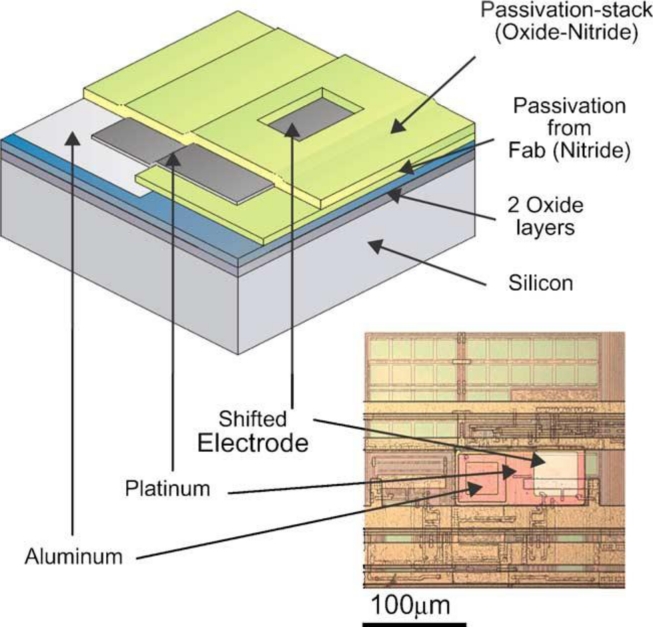
Adaptation of CMOS using photolithographic processing to re-define electrodes using platinum (from [64]). This process requires a microfabrication facility to add additional layers on top of the CMOS IC (and therefore does not meet a low-cost criterion).

An alternative approach to the EOS FET is the use of metal (usually platinum) electrodes based on standard CMOS bondpads (Figure 6) [45,48,60,61].

However, because of the cleft between cell and substrate and the double layer at the solid-solution interface, this approach in fact also leads to a capacitive coupling. However, where the interface is particularly tight (*i.e.*, with a small cleft between cell and substrate), models show that it may be possible to provide a first-order (ohmic) response since the cleft can be modelled as a resistive component that sinks an action potential ion current laterally under the cell into the bulk of the electrolyte [62,63]; however achieving such a small cleft is difficult in practice.

Interestingly, despite the difficulties with both the direct and capacitive coupling methods, there are some similarities in their equivalent circuits: both electrical models include a capacitive cleft and both recording electrodes typically connect to a high impedance FET gate. However, as established in [69], the sensitivity of the metal electrode still out-performs the floating gate EOS FET, with the metal electrode clearly more suitable for recordings from small mammalian neurons.

#### (c) Porous Alumina Based Electrodes

Previous work by the University of Bath and King’s College London was unable to obtain neuronal recordings using standard CMOS aluminium electrodes. The main reason for this was uncontrolled corrosion at the aluminium surface. Recent work has focused on demonstrating that aluminium CMOS microelectrodes can be made both biocompatible and effective by converting them to *porous alumina*. ICs were successfully anodised and their barrier oxides electrochemically thinned, resulting in impedance comparable to the unmodified aluminium and other planar electrodes [65]. The alumina had inter-connected pores, as predicted by the earlier work using Al-Si-Cu coverslips [6]. It was shown that infiltrating the alumina pores with metal further reduced impedance. For applications requiring a planar electrode surface, gold was electrodeposited into the porous alumina to provide a bio-inert surface. For neuronal recording applications that often call for particularly low impedances, the planar gold was coated with platinum-black resulting in a further reduction in impedance to less than 40 kΩ (at 1 kHz) for each 30 μm diameter electrode.

### 4.4. Maximising the Neuronal Recording Signal

**Figure 7.**
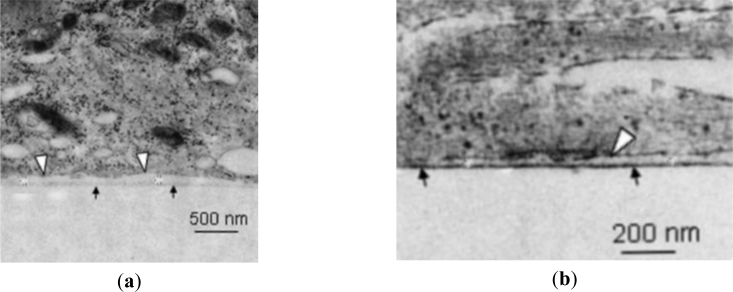
TEM images showing examples of cell-substrate clefts—from [75]. Cells have been fixed and sectioned using a focussed ion beam: (**a**) A platinum substrate (the surface marked with black arrows) was coated with laminin-111 prior to adhesion of chicken embryo neurons. The cleft is between the adhered cell membrane (marked by white arrows) and the platinum surface and was measured to be 27–108 nm; (**b**) L1 Ig6 (the sixth immunoglobulin domain of cell adhesion molecule L1 and known to promote neurite extension) has a lower molecular weight (8 kDa) and is a smaller molecule than laminin-111 (∼800 kDa). Smaller molecules generally result in smaller clefts, as illustrated here by the cleft of 26–79 nm.

The height of the cleft between the cell and electrode is a key parameter that influences the magnitude of extracellular neuronal signals and has therefore been a primary consideration in the development of state of the art neuronal sensors [66]. The use of adhesion proteins has been a main line of investigation as a tool to minimise the cleft by forming tight electrode junctions with the electrode/substrate. It was concluded in [67] that the proteins promote cell adhesion and that the cleft can be minimised using a coating of RGD peptide sequence (RGD = arginine–glycine–aspartic acid (Arg-Gly-Asp)—a laminin and fibronectin fragment). In [68] it was noted that RGD immobilises cells, with the exception of neurons. Polylysine was also discussed as an adhesion molecule but the cleft size was expected to be larger compared to a layer of RGD peptide. In [63] and [69] it was stated that the YIGSR peptide sequence—a laminin fragment—also promotes cell adhesion whilst minimising cleft size (YIGSR = tyrosine–isoleucine–glycine–serine–arginine). Other methods to promote good adhesion include the use of polyethylenimine (PEI) and laminin [70]. Whilst generally successful at producing adhesion, the cleft is wider and so they produce a less efficient electrical interface (Figure 7) (coupled voltage reduces with the square of the cleft distance). A MEMS approach to adhesion has also been investigated whereby a wafer was micromachined to provide pneumatic anchoring of rat cardiomyocytes [71]. This technique has been successfully incorporated into a family of single-use MEAs manufactured by Cytocentrics AG (Germany) [72]. Investigations by [73,74] showed that modification of a silicon surface by patterning (in the range of tens of nanometers to micrometers) can also assist with attachment.

Silicon substrates have been successfully modified by [76] and [77] to produce porous silicon. Scanning electron microscope (SEM) images suggested a tight junction, but these need to be confirmed by electrical characterisations. Unfortunately, the opening of windows to silicon introduces the same drawback as the EOS FET in that ionic contamination of the CMOS logic circuits is likely.

A second key parameter affecting the effective signal of a neuronal recording is the impedance of the electrode. The majority of publications reporting successful recordings from CMOS ICs (c.f. references in Section 4.3 above) use a coating on the electrode of *platinum black* to increase its effective surface area and hence decrease its impedance. This is a well-established method used for electrophysiology and other electrochemistry applications [78]. CMOS IC electrodes have been successfully coated with biocompatible platinum and platinum black [79]: the platinum pads were created by a lithographic patterning step, but the subsequent platinum black was an electrodeposition performed by biasing the stimulation circuitry. The platinum black was shown to reduce successfully the electrode impedance. Interestingly, the benefit arising here from decreased electrode impedance is not intuitive: the improvement comes not from an increase in signal amplitude (the signal magnitude relates to the proportion of an electrode that is covered by a cell, *i.e.*, forming a potential divider. Decreasing the impedance per unit area using platinum black decreases the impedance under the cell but also decreases the impedance to the grounded bulk electrolyte and therefore the platinum black has little direct effect on signal amplitude) but instead from reducing the noise produced by the electrode itself—*i.e.*, the benefit is an improved signal-to-noise ratio at the FET gate input. This occurs since the ‘root mean square’ of the instantaneous noise (r.m.s.) thermal noise (additional sources of noise exist in electrode-electrolyte interfaces [80] but thermal noise dominates in neuronal recording FET-based electrodes), *V*, produced in an electrode is proportional to the root of its resistance, *R*, where, *k* is Boltzmann’s constant, *T* is temperature and *B* is the bandwidth [81,82] (see Equation 1):

(1) $$V=\sqrt{4k\;TBR}$$

**Figure 8.**
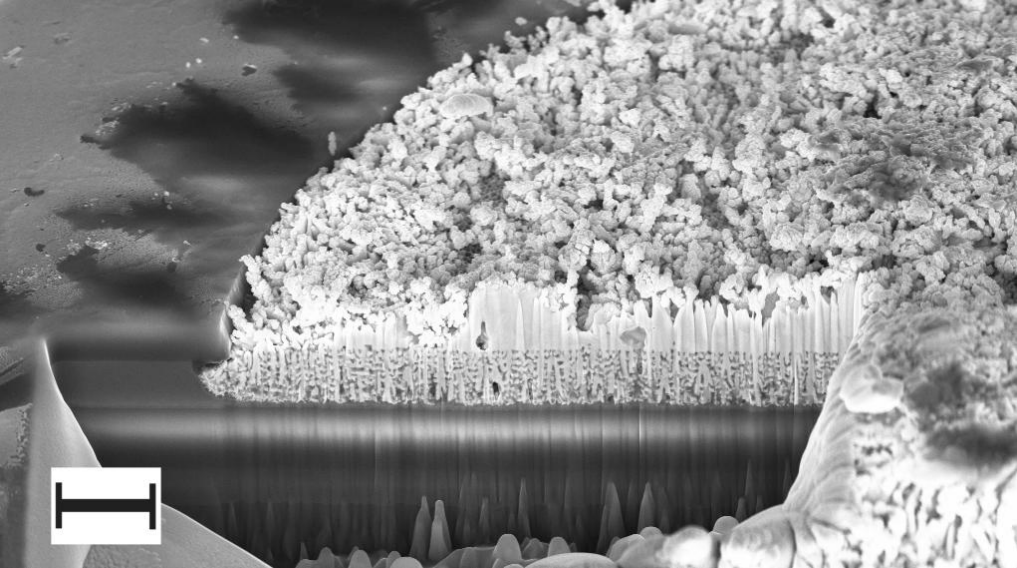
Porous alumina and gold CMOS electrode after deposition of platinum black at 50 mA·cm^−2^. The platinum is within the defined pad area and is flush with the passivation surface. Scale bar is 2 μm.

Commercial MEAs used for mammalian neuronal recordings include the Multi Channel Systems and MED64 (Alpha Med Scientific, Inc.) products. Quality assurance data for MEAs purchased from Multi Channel Systems showed all electrodes had individual impedances of 39 k–41 kΩ at 1 kHz (the characteristic frequency of an action potential), that is in agreement with their documentation claiming 20 k–400 kΩ for their range of titanium nitride electrodes [83]. The MED64 electrodes are claimed to have impedances of 7 k–10 kΩ at 1 kHz [84]. To prepare CMOS IC electrode arrays for neuronal recordings at the University of Bath, it was decided that the 1 MΩ impedance at 1 kHz achieved with the gold deposition alone was unacceptably high. The standard technique of lowering the impedance by coating with platinum black was used, with a target impedance of 40 kΩ (at 1 kHz) for 30 μm diameter electrodes. Figure 8 shows a porous alumina and gold CMOS electrode after deposition of platinum black.

As outlined in Section 1, the potential applications of a silicon-electrode junction are diverse and so the particular type of cell being interfaced is relevant and must be taken into consideration when reviewing recent developments in the literature. For example, many of the published results demonstrating successful recordings from electrically active cells relate not to mammalian neurons but to *human embryonic kidney* (HEK) cells and *cardiomyocytes* [48,62,70,79,85–96]. Such cells may often produce signals of larger amplitude than achievable with mammalian neurons. Therefore, due to the low electrode impedance, cell positioning and small cleft requirements, producing reliable extracellular mammalian neuron recordings using CMOS electrodes is probably one of the most demanding applications. The above review of neuronal recording serves to illustrate the complexity of many CMOS transducers and cell-based biosensor applications where the neurons serve as the bioreceptor.

## 5. CMOS Neural Interfaces

There are many areas of biomedicine that are driving developments in the stimulation and recording of neuronal electrical activity. Applications are primarily drug discovery pharmacology, neural interface systems, cell-based biosensors and systems to assist in the understanding of neural network behaviour. Techniques are available that span the scale of (spatial) resolution: whole brain imaging is possible through methods such as *electroencephalography* (EEG), *positron emission tomography* (PET) and *functional magnetic resonance imaging* (fMRI) [97]. Populations and networks of neurons can be observed using voltage-sensitive optical dyes that provide response times (temporal resolution) usually into the millisecond range, although recent progress has extended the resolution into the sub-millimetre range [98]. However, dyes can be toxic, the dye metabolites can be toxic, or strong illumination can cause photodynamic damage [99]. Single neuron recordings may be non-invasive but provide only very limited information from a small region of space, typically 10–50 μm [100]. As an extension to the methods of Hodgkin and Huxley, the patch clamp retains the benefit of excellent temporal resolution, effectively unlimited by the fast response times of electronic measurement instruments. The patch clamp is therefore an excellent method for electrical stimulation and recording of single cells but, being an invasive method (with the clamp damaging the cell membrane), the recording duration is usually limited to a few hours.

**Figure 9.**
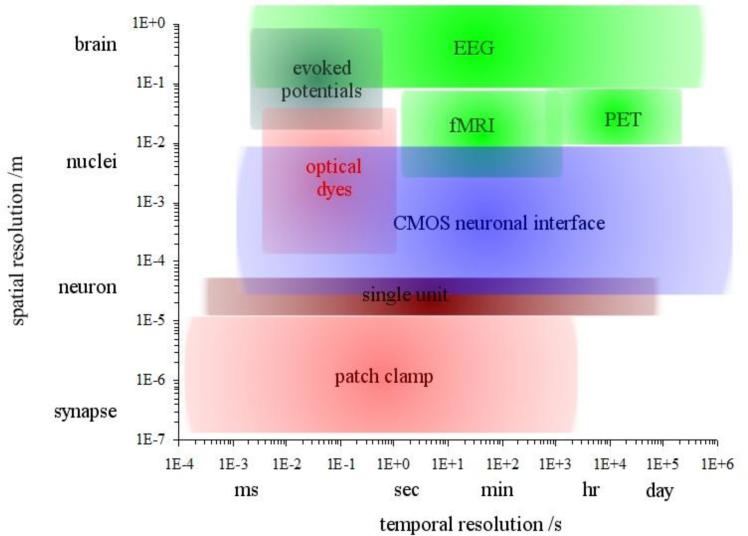
Map of imaging techniques on a spatial-temporal plane (adapted from [100]).

It can be seen from Figure 9 that there is a lack of techniques that provide a spatial resolution enabling recording from one or more neurons but with a temporal resolution that allows action potential recordings (sub-millisecond) for many days. This is important for monitoring a range of biological processes in single neurons or networks such as drug tolerance, neurotoxicity, neurodegradation, network development and activity, including learning, memory and circadian rhythm. This gap in techniques can be filled by non-invasive extracellular electrodes, and the ability to lay out an array of electrodes on a surface for greater spatial coverage makes IC technology a good candidate for this purpose.

### 5.1. The Electronic-Neuronal Interface: Commercial MEAs

This section discusses MEAs for neuronal recording and stimulation applications where the electrode is acting as a transducer. Although generally suffering from limited spatial resolution and expensive to manufacture, passive MEAs have been manufactured commercially for some time. As a result, as already noted, much work was begun during the 1990s in order to leverage the potential benefits of planar semiconductor technologies—primarily CMOS—offering a potentially cheap source of electrodes, integrated signal processing and increasingly excellent spatial resolution (with on-chip signal multiplexing and processing, large CMOS arrays may be formed with spatial resolution limited by the cell culture density rather than the IC technology. For example, a 128 × 128 array of 10 μm diameter electrodes at 50 μm spacing would be a resolution of 4 × 10^8^ m^−2^). Early investigations, especially by groups such as Fromherz *et al.* at the Max Planck Institute of Biochemistry identified the adherence of biological cells to the IC electrodes as a major challenge [101]. Progress has been slow as the factors influencing cell-substratum adhesion are complex.

**Figure 10.**
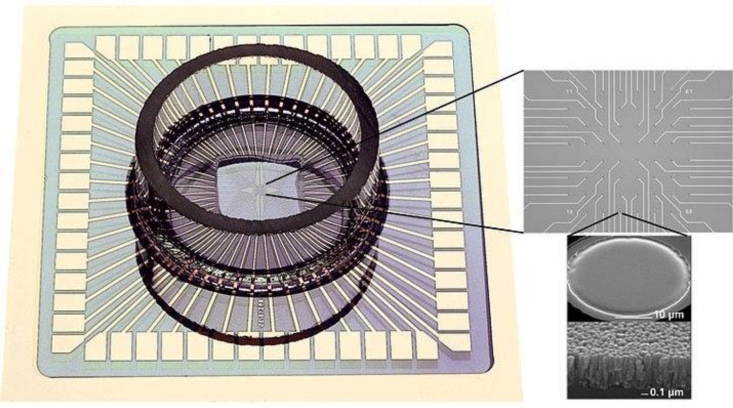
A Multi Channel Systems MEA. The magnified images show the array of 64 electrodes and further SEM images showing a single electrode and its surface. The lower image shows the high surface area dendritic TiN (from [102]).

The principal manufacturer of MEAs is presently Multi Channel Systems (MCS) GmbH (Germany), supplying a range of MEAs with planar microelectrodes, typically an array of 60 recording electrodes, plus an optional large planar stimulation electrode [102]. MCS have developed a comprehensive set of amplification, data acquisition and data analysis tools to support their MEAs product line. MCS have identified that low impedance electrodes are necessary in order to achieve acceptable signal-to-noise ratios and they have therefore developed a high surface area titanium nitride (TiN) electrode which typically achieves an impedance of only 40 kΩ at 1 kHz for a circular electrode of 30 μm diameter (Figure 10). Whilst the electrodes are high performance, their durability is low: MCS state that the electrodes are re-usable but cleaning is difficult as the electrodes cannot be touched with cotton buds, etc, due to the fragility of the TiN. The electrodes also seem to degrade in ambient conditions and therefore might have a useful maximum lifetime of one year. Other suppliers of passive MEAs use conventional platinum and platinum black electrodes, probably due to their improved robustness. Ayanda Biosystems SA (Switzerland), manufacture MEAs with a footprint compatible with the MCS data acquisition systems [103].

**Figure 11.**
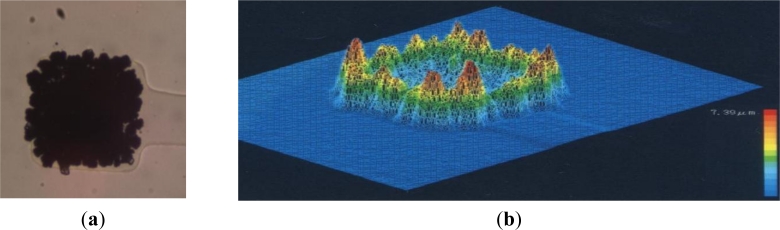
MED64 MEA: (**a**) a single 50 × 50 μm electrode showing copious platinum black deposition used to achieve a low impedance; (**b**) profile of a MED64 electrode showing tall (7.39 μm) dendritic growth, mainly at the periphery of the electrode (from [105]).

Ayanda produce square (40 × 40 μm) platinum electrodes with typical impedances of 400–600 kΩ (at 1 kHz). In partnership with Bio-Logic SAS (France), Ayanda also produce passive MEAs with up to 256 electrodes [104]. Alpha Med Scientific, Inc.(Japan), manufacture the MED64 system which comprises 64 electrode MEAs and supporting data acquisition and analysis instrumentation [105]. All three manufacturers use glass substrates which have the benefit of allowing imaging of the cells using phase contrast microscopy. MEAs can be supplied optionally with indium tin oxide (ITO) tracks that are transparent at visible wavelengths and therefore further improve the clarity of optical microscopy. This is not possible with CMOS as the silicon substrate is opaque. The MED64 system uses square electrodes of 50 × 50 μm coated with platinum black to achieve a low impedance of only 7–10 kΩ (at 1 kHz). It should be noted that this low impedance is achieved at the expense of the platinum black creating tall (7.39 μm) dendritic growths above the substrate surface (Figure 11). It is questionable whether the body (soma) of dissociated neurons will readily cover such a tall feature and instead the cells may prefer to adhere only to the substrate.

A recent entrant into the MEA market is the company 3Brain [106], a spin-out of CSEM (Centre Suisse d’Electronique et de Microtechnique) and represents a first for the penetration of CMOS technology into the MEA market.

**Figure 12.**
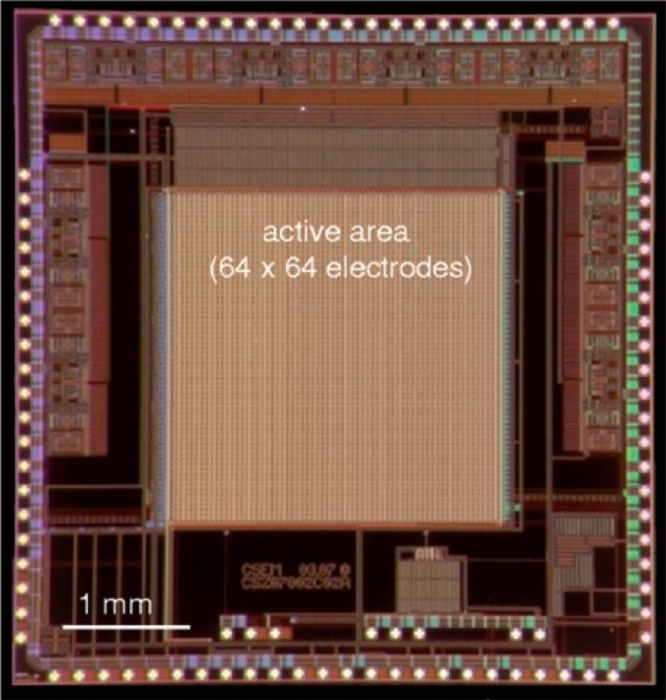
A CMOS-based sensor for high spatio-temporal resolution electrophysiology, produced by 3Brain. The device comprises an array of 4096 electrodes, each 21 μm × 21 μm. The post-processing and electrode surface material is undisclosed (from [44]).

The 3Brain APSMEA chip (Figure 12) uses CMOS to form an array of 4,096 electrodes for high spatial resolution electrophysiology. At approximately £140, it appears competitively priced against the Multi Channel Systems passive MEA (£250–300 each). Each electrode is 21 μm × 21 μm with a pitch of only 42 μm. The IC includes on-chip address decoders and signal processing, and claims high sensitivity and low-noise [44]. Details of the CMOS post-processing and the electrode surface are undisclosed [44,107] and therefore a detailed appraisal of their process is not possible here.

### 5.2. Other Commercial CMOS-Based Biosensors

Beyond neuronal interfaces, commercial CMOS-based products are presently few. However, new products are emerging, but are mainly beyond the scope of this article (*i.e.*, not cell-based), such as the DNA sequencing chip from Ion Torrent (now part of Life Technology Corp.) [108]. However even here, the use of CMOS requires additional layers to be deposited on the top of the CMOS and patterned using photolithography to provide biocompatibility. Similarly, CustomArray Inc. also uses CMOS in their DNA synthesis chips where photolithographic post-processing is required to form the electrode array [109]. Bionas use CMOS ICs in a 96-well plate format [110]. This seems to represent the first commercial product using CMOS in such a format and is an indication of what it likely to become a considerable focus for this technology in the future. However, the Bionas ICs require considerable photolithographic custom-/post-processing steps to integrate the various type of sensor (pH, oxygen consumption and cell-substrate impedance).

## 6. CMOS Packaging Technology

A biosensor generally requires some form of carrier or package to support and position its transducer. However, a key requirement of most biosensors is that the active area of the transducer is exposed to the external environment. This is different from most other forms of IC where input is through the package external electrical connections (e.g., leads or solder bumps) and these form a seal between the internal device and the environment. IC sensors may, for example, measure temperature, pressure, acceleration or light intensity (e.g., a photodiode or camera array) but these ICs can usually be sealed from their external environment (e.g., using a transparent window for light sensors) [111]. The micro-electromechanical systems (MEMS) market initially leveraged the semiconductor industry for both substrate and packaging technology, but the specific requirements of MEMS applications have more recently driven the design of new specialised package types [112]. Table 2 summarises some of the challenges and illustrates why the biosensor market struggles to utilise high-volume semiconductor assembly methods.

However, there are still many applications where suitable solutions are sparse, especially for biosensors integrating MEMS-based transducers (‘Bio-MEMS’) [114]. Applications based on semiconductor ICs are such an example, requiring demanding packaging solutions not readily available commercially. For instance, a cell-based biosensor with an IC transducer raises unique difficulties where the transducer on the semiconductor die must contact cell culture media but simultaneously provide biocompatible electrical and chemical isolation from the bondpads and bondwires at the edge of the die [115].

**Figure 13.**
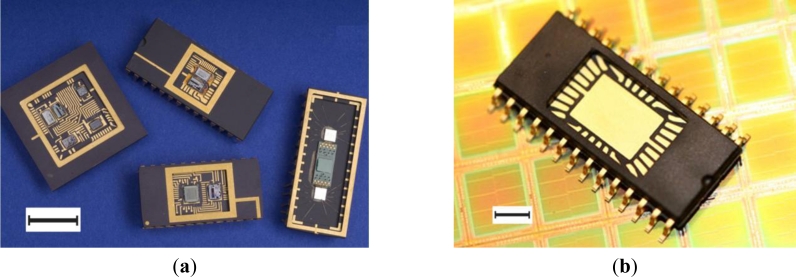
Example packaging technologies frequently adapted for MEMS applications. (**a**) multi-chip-module, ‘system-on-chip’ (from [117]; scale bar is 5 mm); (**b**) open-cavity package, shown without die and bondwires (from [116]; scale bar is 1 mm).

Flexible assembly processes can accommodate the bonding of multiple components, including die and discrete components onto a single substrate to a form multi-chip-module (MCM, [Figure 13(a)]). Similarly, components can be assembled into a single package resulting in a system-on-chip (SoC). Standard packages can be used as a basis for the encapsulation, such as ceramic dual-in-line pin (DIP), plastic DIP (PDIP), QFN, QFP, SOIC and SSOP outlines [116]. As an alternative to silicon, lower-scale integration is achievable on a range of flexible (polymer) or rigid (e.g., glass) substrates by depositing layers to form thin film transistors (TFTs).

With the industry in its infancy, several prototyping solutions for IC biosensors have been developed by researchers to meet their specific needs [118–120], but presently there are no standards. Companies such as Sempac, U.S. [115] and Quik-Pak, U.S. [121] provide custom packaging solutions [Figure 13(b)] but these are based on semiconductor package outlines and are based on materials which may not be suitable for the biosensor applications (e.g., moulding compound biocompatibility, flexible substrates for use *in vivo*).

## 7. Obstacles to Commercialisation

There are also generic barriers to commercialisation for all CMOS-based biosensor applications that are now discussed. Commercial transducers based on proprietary substrate technologies are not readily scaled to applications requiring a large numbers of electrodes, such as drug discovery and multiple electrode arrays for neuronal recordings. However, more scalable technologies such as CMOS have yet to demonstrate reliable operation as MEAs. The criteria for success are the ability to culture neuronal cells on electrochemically and electronically stable CMOS electrodes, maintain cell health (vitality) through good biocompatibility and to demonstrate the recording of action potentials.

Through continuous development of the CMOS interface, particular constraints and techniques are emerging that may lead to successful electrode products. These are summarised in Table 3. The *constraints* are effectively the requirements of a commercial interface whilst the *emerging characteristics* are defined as the techniques being pursued by a number of research groups.

## 8. Conclusions, Outlook and Future Work

IC technology is readily available as a biosensor transducer in its ubiquitous form as *complementary metal oxide semiconductor* (CMOS). However, to create a suitable interface to biological cells the transducer must include electrodes that are non-invasive and biocompatible—requirements that CMOS does not naturally meet. Researchers have therefore modified CMOS ICs by applying additional layers, but the only method proven to establish biocompatibility requires the use of microfabrication equipment in a semiconductor cleanroom. This is suitable for ICs manufactured for research purposes but, due to the high cost of this approach, the economies of scale provided by CMOS are lost. If CMOS-based biosensors are to be commercialised, then a low-cost method to modify the technology is required.

Cell-based biosensor research has taken the first steps towards commercialisation but further work is needed to demonstrate CMOS technology in a commercial context. Firstly, packaging will remain an issue: either new methods will be required (e.g., microtitre plates with ICs in each well) or the partial encapsulation process will need refining. Considerable effort will be required in terms of ‘design for manufacture’ as the CMOS IC must be capable of supporting the post-processing (e.g., anodisation and electrodeposition processes) which must integrate with other IC functional blocks such as amplifiers, data processing and communications circuits. Subsequently, once an IC design and post-processing steps are finalised, the manufacturability (yield, process parameters) and reliability will need optimising. Industry-standard semiconductor reliability tests can be adopted for this purpose, supplemented by additional tests specific to biosensors such as biocompatibility testing. The process must also be future proof against fast moving semiconductor technology and it will be necessary for the post-processing methods to periodically transition to newer CMOS fabrication processes. From a commercial viewpoint, a more thorough understanding of projected production costs must be performed and a business risk assessment made against competing technologies. For example, bench top processing of ICs seems intrinsically cost effective, but scaling to the high volumes of a production line has yet to undergo rigorous business analysis. In addition, if multiple ICs are to be integrated into the wells of microtitre plates, then acceptable production yield will need to be proven.

The adoption of suitable IC post-processing may therefore foster the commercialisation of CMOS cell-based biosensors in drug discovery, neuroprosthetics, and environmental applications and enable affordable research tools for bioscience.

## Figures and Tables

**Table 1.** Major worldwide foundries (for digital and analogue ICs). The list includes companies that manufacture primarily their own IC designs (known as ‘integrated device manufacturers’) and ‘dedicated foundries’ that manufacture for fabless companies.

Analog Devices
Atmel
AMS
Chartered Semiconductor (Chartered)
Elpida
Fairchild
Freescale
Fujitsu
Global Foundries
Grace Semiconductor, China
HHNEC (NEC)
Hynix
IBM
IMEC
Numonyx (now Micron)
Infineon
Intel
International Rectifier
Matsushita/Panasonic
Microchip
Micron
Mitsumi
National Semi
NXP (formerly part of Philips)
ON Semiconductor
Powerchip
ProMOS
Qimonda (formerly part of Infineon)
Renesas (formerly NEC Electronics America)
ROHM (OKI Semiconductor)
Samsung
Sanyo Semiconductor (USA) Corp.
Semiconductor Manufacturing International Corporation (SMIC)
Sony
Spansion
STMicroelectronics (STMicro)
Taiwan Semiconductor Manufacturing Company (TSMC)
TECH Semiconductor (TECH Semi)
Texas Instruments (TI)
Toshiba
TowerJazz Semicondcutor
United Microelectronics Corporation (UMC)
Vangaurd International Semiconductor

**Table 2.** Summary of MEMS packaging requirements *versus* standard integrated circuit packaging (adapted from [113]).

**Bio-MEMS Applications**	**Standard Integrated Circuits**

Often involve moving solids or fluids	Stationary thin solid structures
Require integration of microstructures with microelectronics	No such integration is required
Perform a variety or functions of biological, chemical, optical and electromechanical nature	Transmit electrical signals only
Many components are required to interface with working media and hostile environments	Integrated circuit die are protected from working media by encapsulation
Fewer electrical connections and leads	Large number of electrical connections and leads
Lack of engineering design methodology and standards	Well-established design methodology and standards
Packaging technology is in its infancy	Mature packaging technology and clearly defined roadmaps
Assembly is primarily manual	Highly automated assembly techniques available
Lack of quality and reliability testing standards and test facilities	Mature standards and established quality and reliable testing facilities
Distinct manufacturing techniques for each application	Manufacturing techniques are proven and well documented
No industrial standards to follow in design, manufacture, packaging and testing	Well-established methodologies and procedures

**Table 3.** Themes for commercially-viable CMOS neuronal interfaces.

**Constraints (for commercially-viable solutions)**	**Emerging Characteristics**

use of standard CMOS technologies(mature processes: low cost; high availability)	bondpad modification using plating

	high spatial resolution (for MEAs) using addressable sensor arrays
no complex post-processing of ICs (e.g., no additional lithography)	minimised cleft by using specific adhesion layer peptides
useful lifetimes for long *in vitro* assays or long-term (chronic) implantation	improved packaging based on MEMS technology

	robust signal amplification using very low-noise amplifier designs
